# Supplementary material for: Experimental verification of field-enhanced molecular vibrational scattering at single infrared antennas
Source: Nat Commun. 2024 Aug 8;15:6760. doi: 10.1038/s41467-024-50869-x (PMC11310513; doi:10.1038/s41467-024-50869-x)
Supplement: Supplementary file 1 — Supplementary Information [file 41467_2024_50869_MOESM1_ESM.pdf]

Supplementary Information to

Experimental verification of field-enhanced molecular vibrational scattering at single infrared antennas

Divya Virmani,[1] Carlos Maciel-Escudero,[1,2] Rainer Hillenbrand,[1,3,4*] and Martin Schnell[1,3*]

[1] *CIC nanoGUNE BRTA, 20018 Donostia-San Sebastián, Basque Country, Spain*

[2] *Materials Physics Center, CSIC-UPV/EHU, 20018 Donostia-San Sebastián, Spain*

[3] *IKERBASQUE, Basque Foundation for Science, 48013 Bilbao, Basque Country, Spain*

[4] *Department of Electricity and Electronics, UPV/EHU, 20018 Donostia-San Sebastián, Spain*

**r.hillenbrand@nanogune.eu*

**schnelloptics@gmail.com*

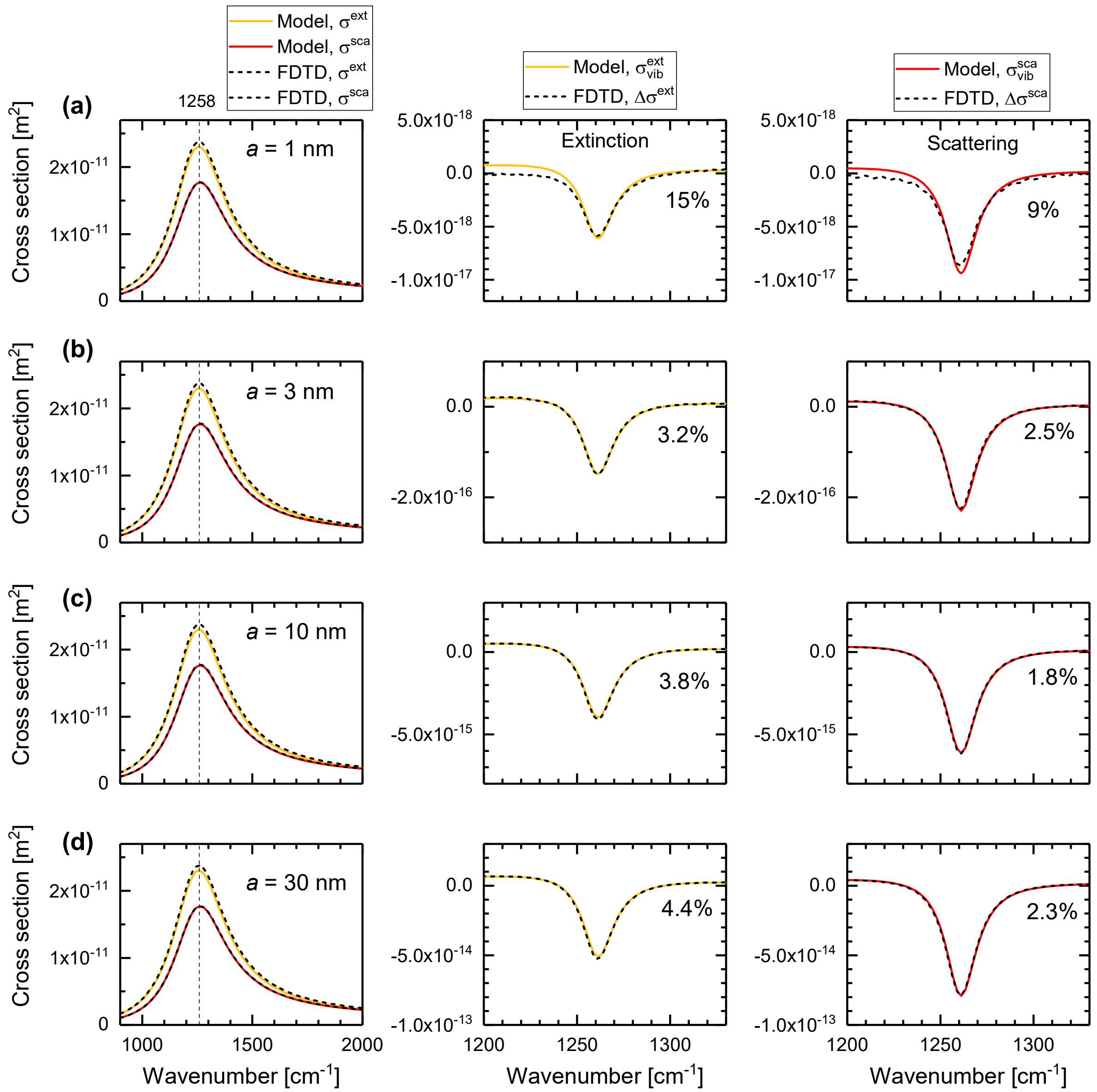

Supplementary Figure 1: Numerical validation of the scattering model. Variation of particle radius. (a)-(d) An IR-resonant Au nanorod of length, $L = 3.19$ μm, couples to a small particle with a vibrational resonance at 1,258 cm^{-1}. This is the same calculation as in Fig. 1(b) in the main text, but here the particle radius is varied to $a = 1$ nm, 3 nm, 10 nm, 30 nm is assumed. First column: Modeled (Eqs. (11),(12) in the main text) and numerically calculated extinction and scattering cross section, σ^{ext} and σ^{sca}. Second and third columns: Spectral signature of the molecular vibration in the extinction and scattering cross section as obtained with the scattering model, $\sigma_{\text{vib}}^{\text{ext}}$ and $\sigma_{\text{vib}}^{\text{sca}}$ (Eq. (13) in the main text), compared to numerical calculations, $\Delta\sigma^{\text{ext}}$ and $\Delta\sigma^{\text{sca}}$. The number given in the plots is the maximum relative error between calculation and the scattering model. Note larger error observed with small particle diameter $a = 1$ nm is attributed to numerical error.

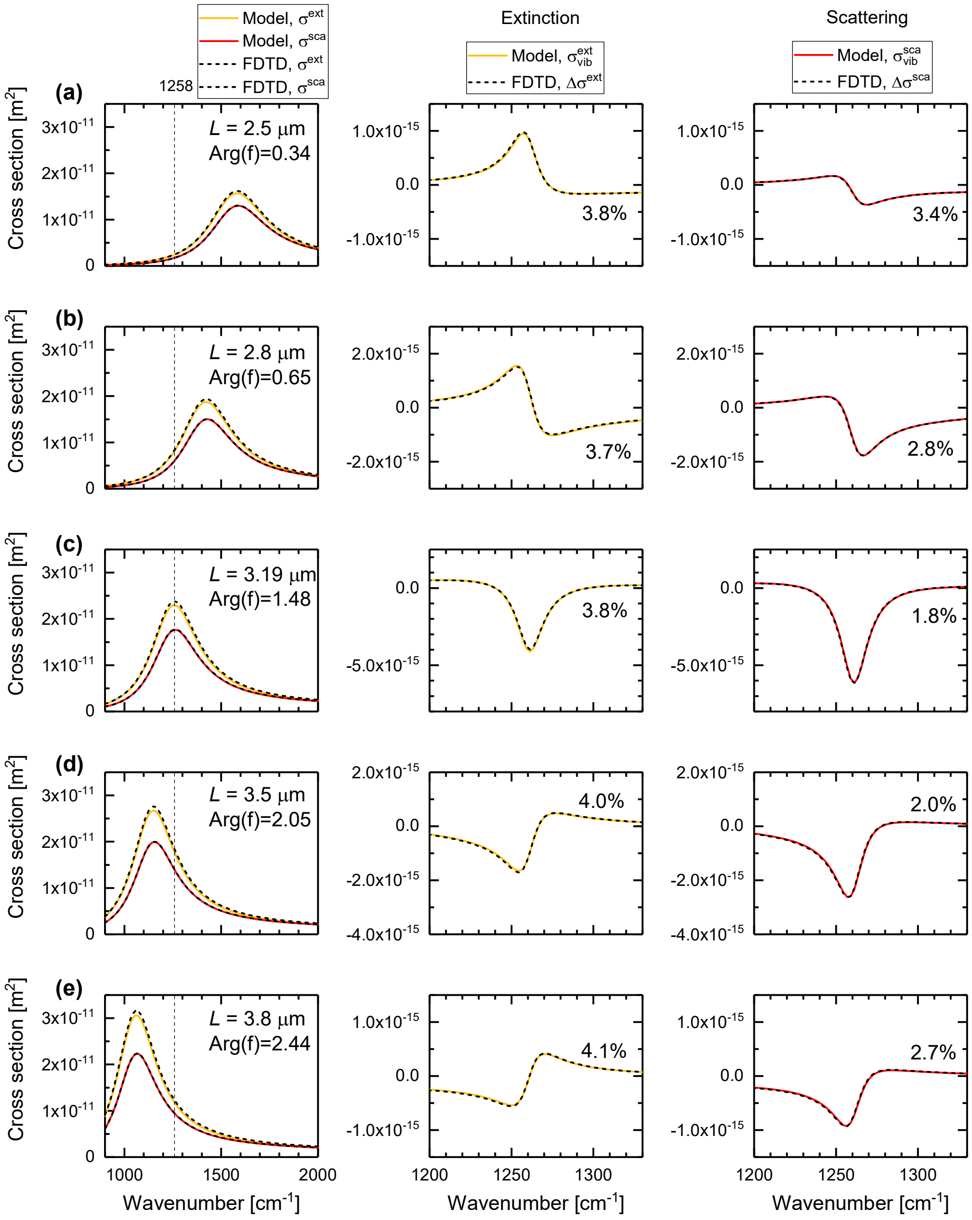

Supplementary Figure 2: Numerical validation of the scattering model. Variation of antenna length. (a)-(e) An IR-resonant metal couples to a small particle with a vibrational resonance at 1,258 cm^{-1}. This is the same calculation as in Fig. 1(b) in the main text, but here a particle radius of 10 nm is assumed and the nanorod length is varied to $L = 2.5\ \mu\text{m}, 2.8\ \mu\text{m}, 3.19\ \mu\text{m}, 3.5\ \mu\text{m}, 3.8\ \mu\text{m}$. First column: Modeled (Eqs. (11),(12) in the main text) and numerically calculated extinction and scattering cross section, σ^{ext} and σ^{sca}. $\text{Arg}(f)$ specifies the phase (in radians) of the field enhancement f provided by the antenna, evaluated at the vibrational resonance at 1,258 cm^{-1}, which controls the observed line shapes. Second and third columns: Spectral signature of the molecular vibration in extinction and scattering cross section as obtained with the scattering model, $\sigma^{\text{ext}}_{\text{vib}}$ and $\sigma^{\text{sca}}_{\text{vib}}$ (Eq. (13) in the main text), compared to numerical calculations, $\Delta\sigma^{\text{ext}}$ and $\Delta\sigma^{\text{sca}}$. The number given in the plots is the maximum relative error between calculation and the scattering model.

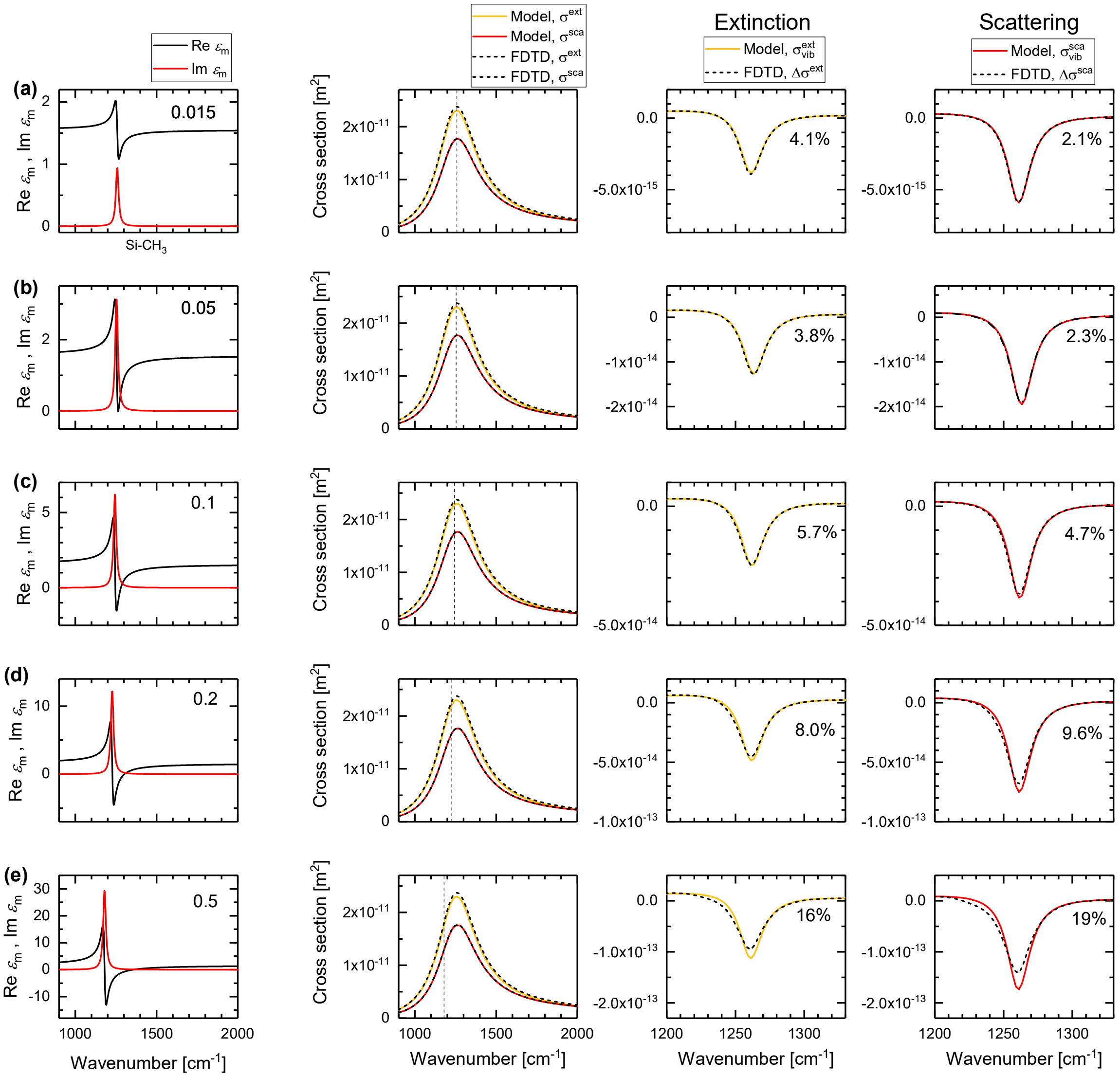

Supplementary Figure 3: Numerical validation of the scattering model. Variation of oscillator strength. (a-e) An IR-resonant metal antenna couples to a small particle. This is the same calculation as in Fig. 1(b) in the main text, but here a particle radius of 10 nm is assumed and further the particle vibrational resonance is modeled with the following oscillator strengths: $\varepsilon_{\mathrm{Lorentz}} = 0.015,\ 0.05,\ 0.1,\ \ 0.2,\ 0.5$ and $\omega_{\mathrm{Lorentz}} = 1{,}258,\ 1{,}254,\ 1{,}244,\ 1{,}227$ and $1{,}170\ \mathrm{cm}^{-1}$. First column: Object permittivity, $\varepsilon_{\mathrm{O}}(\omega)$. Second column: Modeled (Eqs. (11),(12) in the main text) and numerically calculated extinction and scattering cross section, σ^{ext} and σ^{sca}. Third and fourth columns: Spectral signature of the molecular vibration in extinction and scattering cross section as obtained with the scattering model, $\sigma_{\mathrm{vib}}^{\mathrm{ext}}$ and $\sigma_{\mathrm{vib}}^{\mathrm{sca}}$ (Eq. (13) in the main text), compared to numerical calculations, $\Delta\sigma^{\mathrm{ext}}$ and $\Delta\sigma^{\mathrm{sca}}$. The number given in the plots is the maximum relative error.

	Nanoparticle radius a	1 nm	3 nm	10 nm	30 nm
Small particle	$\sigma_0^{\text{sca}}\ [m^2]$	$2.3 \cdot 10^{-31}$	$1.69 \cdot 10^{-28}$	$2.32 \cdot 10^{-25}$	$1.69 \cdot 10^{-22}$
	$\sigma_0^{\text{abs}}\ [m^2]$	$2.2 \cdot 10^{-21}$	$6.00 \cdot 10^{-20}$	$2.22 \cdot 10^{-18}$	$6.00 \cdot 10^{-17}$
Field-enhcd.	$\lvert f\rvert^4\sigma_0^{\text{sca}}\ [m^2]$	$1.7 \cdot 10^{-24}$	$1.05 \cdot 10^{-21}$	$7.58 \cdot 10^{-19}$	$1.25 \cdot 10^{-16}$
	$\lvert f\rvert^2\sigma_0^{\text{abs}}\ [m^2]$	$6.1 \cdot 10^{-18}$	$1.49 \cdot 10^{-16}$	$4.01 \cdot 10^{-15}$	$5.15 \cdot 10^{-14}$
Extinction	FDTD $\Delta\sigma^{\text{ext}}\ [m^2]$	$-5.8 \cdot 10^{-18}$	$-1.50 \cdot 10^{-16}$	$-4.12 \cdot 10^{-15}$	$-5.28 \cdot 10^{-14}$
	Model $\sigma_{\text{vib}}^{\text{ext}}\ [m^2]$	$-5.9 \cdot 10^{-18}$	$-1.49 \cdot 10^{-15}$	$-3.99 \cdot 10^{-15}$	$-5.12 \cdot 10^{-12}$
Scattering	FDTD $\Delta\sigma^{\text{sca}}\ [m^2]$	$-8.6 \cdot 10^{-18}$	$-2.26 \cdot 10^{-16}$	$-6.16 \cdot 10^{-15}$	$-7.89 \cdot 10^{-14}$
	Model $\sigma_{\text{vib}}^{\text{sca}}\ [m^2]$	$-9.2 \cdot 10^{-18}$	$-2.29 \cdot 10^{-15}$	$-6.15 \cdot 10^{-15}$	$-7.91 \cdot 10^{-12}$
Enh. Factors	F^{ext}	$2.9 \cdot 10^{13}$	$1.04 \cdot 10^{12}$	$2.04 \cdot 10^{10}$	$3.61 \cdot 10^{8}$
	F^{sca}	$4.5 \cdot 10^{13}$	$1.60 \cdot 10^{12}$	$3.13 \cdot 10^{10}$	$5.54 \cdot 10^{8}$

Supplementary Table 1: Comparison between molecular scattering and molecular absorption. *Small particle* scattering and absorption cross sections of a spherical nanoparticle with a vibrational resonance as obtained analytically with $\sigma_0^{\text{sca}} = \frac{k^4}{6\pi}\lvert\alpha_0\rvert^2$ and $\sigma_0^{\text{abs}} = k\text{Im}\{\alpha_0\}$, respectively, where k is the magnitude of the free-space wavevector and α_0 is the object polarizability. *Field-enhanced* scattering and absorption cross sections of the small particle, $\lvert f\rvert^4\sigma_0^{\text{sca}} = \frac{k^4}{6\pi}\lvert f\rvert^4\lvert\alpha_0\rvert^2$ and $\lvert f\rvert^2\sigma_0^{\text{abs}} = k\lvert f\rvert^2\text{Im}\,\alpha_0$. *Extinction & Scattering*: Numerically calculated (FDTD) spectral signature of the molecular vibration, $\Delta\sigma = \sigma - \sigma_{\text{bkg}}$, and modeled spectral signature, $\sigma_{\text{vib}}^{\text{ext}}$ and $\sigma_{\text{vib}}^{\text{sca}}$ (Eq. (13) in the main text), in the SEIRA extinction and scattering cross section. *Enhancement factors*: Combined enhancement provided by interferometric and field enhancement of molecular scattering in SEIRA extinction and scattering, F^{ext} and F^{sca} (Eqs. (15),(16) in the main text). All data are evaluated at the vibrational resonance at 1,258 cm^{-1}. The corresponding spectra are shown in Supplementary Fig. 1.

Supplementary Note 1 – Numerical Calculations describing the Near-Field Experiment

We provide details on the numerical calculations presented in Figs. 3, 5 in the main text. We considered rectangular shaped Au nanorods (the antenna) of dimension L x 250 nm x 60 nm, where length L is variable as stated in Fig. 3(b) in the main text, while width and height were common for all antennas and were determined from the AFM topography data of the nanorods used in the experiment. The nanorod length L was adjusted to fit the experimental spectral in Fig. 3(a) in the main text. The nanorod is supported on a CaF_2 substrate. In the experiment, the AFM tip was a pyramidically-shaped metallic structure with a nominal height in the range of 10 to 15 µm. The AFM tip was covered by a thin PDMS layer, a well known contamination of commercial AFM tips[5,6]. In the calculation, we approximated the AFM tip by a core-shell nanoparticle, where the radius of the Au core was assumed to be equal to the radius of the tip apex of the AFM tip, that is 50 nm. We additionally assumed a thin PDMS shell to mimic the PDMS contamination of the experimental AFM tip. To match the magnitude of the spectral signature observed in the experiment, the thickness of the PDMS shell was chosen to be 10 nm and further the tapping amplitude was adjusted to 80 nm. We used tabulated values for the permittivity of Au[1], PDMS[7] and CaF_2[1]. In Fig. 5 in the main text, we followed the same approach for the gap antenna by considering two rectangular shaped Au nanorods (the antenna) of dimension L x 250 nm x 60 nm and separated by a 100 nm gap with the AFM tip being placed in the gap.

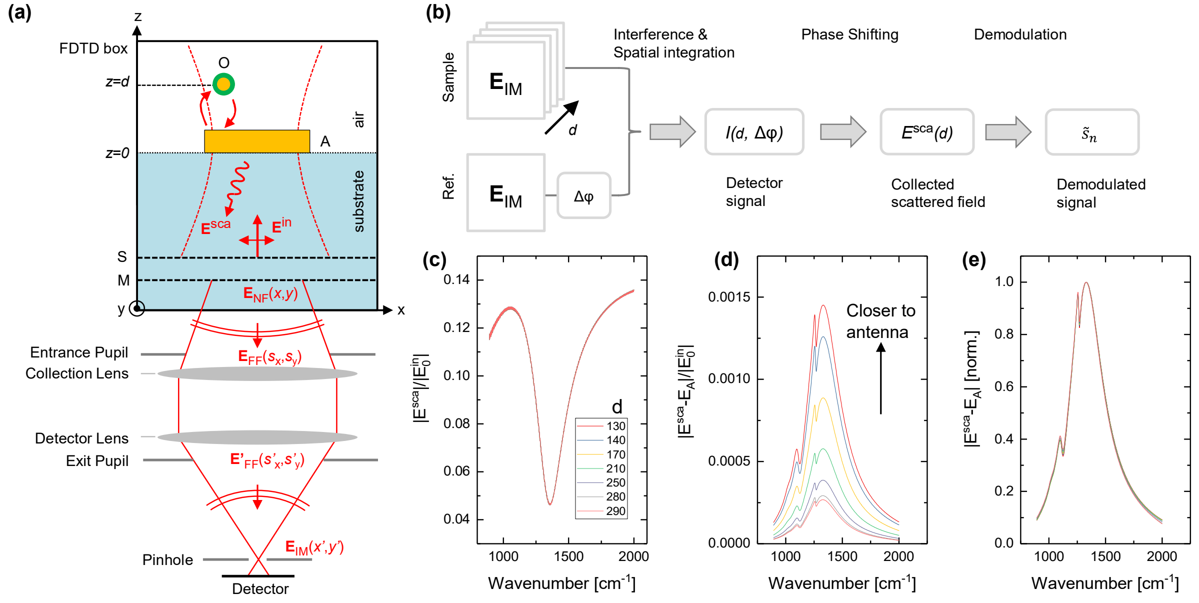

Supplementary Figure 4: Numerical calculation of the near-field experiment. (a) Numerical model which describes the near-field interaction between the antenna (A) and core-shell nanoparticle (O) using the Finite-Difference Time-Domain (FDTD) method, but also takes into account the optical apparatus to accurately describe the light collection. (b) Illustration of the processing chain where the images obtained from the FDTD simulations, $\mathbf{E}_{\mathrm{IM}}(x', y')$, are used to produce a numerically calculated near-field signal, $\tilde{s}_n = s_n e^{i\varphi_n}$, including the aspect of demodulation. (c-e) Illustration of the calculated fields before demodulation is applied. (c) Calculated reflected signal, E^{sca}, for the 2.5 µm long nanorod in Fig. 3 in the main text for a few selected values for the antenna-nanoparticle distance, d. (d) Differential spectra, $E^{\mathrm{sca}} - E_{\mathrm{A}}$, as obtained from (c). (e) Normalized differential spectra as obtained from (d).

The scattered signal of the antenna-nanoparticle system was calculated as follows (solid lines in Fig. 3(b,g) in the main text). We based the numerical calculations on the algorithm described by Çapoğlu et al. [8] for implementing a virtual imaging system on a computer and adapted it to describe the specifics of our near-field experiment in Fig. 3 in the main text. In detail, nanorod (antenna) and core-shell nanoparticle (object) were illuminated with a focused Gaussian beam as injected with source S inside the FDTD box (Supplementary Fig. 4(a)). The optical response of this system was obtained by rigorously solving Maxwell's equation using the FDTD method. The near-field of the antenna-nanoparticle system, $\mathbf{E}_{\mathrm{NF}}(x, y)$, was recorded with monitor M inside the FDTD box and then subsequently propagated to the far field using a near-field-to-far-field transform to obtain the scattered field from the antenna-nanoparticle system, $\mathbf{E}_{\mathrm{FF}}(s_x, s_y)$. Scattering outside the objective NA was truncated and the remaining field was refocused onto the image (detector) plane, yielding electric field $\mathbf{E}_{\mathrm{IM}}(x', y')$. For simplicity, a magnification of $M = 1$ was assumed.

Supplementary Figure 4(b) illustrates the data processing flow. To simulate the signal demodulation used in s-SNOM, a series of simulations was run where the antenna-nanoparticle distance, d, was varied linearly in steps of 5 nm, yielding a total of 35 electric field maps $\mathrm{E}_{\mathrm{IM}}(x', y')$, one for each position of the nanoparticle. Note that a fixed mesh refinement covering the nanorod (antenna) and the core-shell nanoparticle (object) of 5 nm was assumed for all positions of the core-shell nanoparticle (to avoid any residual effects caused by different mesh configurations in the demodulation process) and further a minimum distance of 2 mesh cells between nanorod and core-shell nanoparticle was assumed (to avoid erroneous results when the core-shell nanoparticle and nanorod are in contact). These field maps, E_{IM}, were then processed following the procedure from ref. [9]. First, interferometric detection was implemented by calculating the field of the reference beam at the image plane, $\mathbf{E}_{\mathrm{IM}}^{\mathrm{ref}}$, as obtained by reflecting the focused beam in the simulation at a metal interface. Phase-shifting interferometry was applied to resolve the collected scattered signal, E^{sca}, in amplitude and phase. Supplementary Fig. 4(c) exemplarily shows the collected scattered signal, E^{sca}, for a few selected positions of the core-shell nanoparticle. The antenna resonance is clearly visible but appears as a negative peak owing to interference with the reflected light off the substrate surface. For clarity, we remove the reflection off the substrate surface and the static antenna scattering signal, E_{A}, by running a simulation where the nanoparticle is removed. Supplementary Fig. 4(d) shows the resulting differential spectra, $\Delta E^{\mathrm{sca}} = E^{\mathrm{sca}} - E_{\mathrm{A}}$, revealing the spectral signature of PDMS. Supplementary Fig. 4(e) shows the normalized differential spectra, ΔE^{sca}, showing that the spectral shape of ΔE^{sca} is nearly constant as the nanoparticle approaches the antenna, confirming that modulation of the nanoparticle-antenna distance, d, only enters as a modulation of the field enhancement, $f = f(d)$, but does not affect the spectral shape of the nanoparticle-antenna coupling, $\mathbf{E}_{\mathrm{AOA}}$, as expected[10]. We then constructed the sinusoidal motion of the AFM tip in the experiment, $d = d_0 + \Delta d \cos \Omega t$ (where Δd is the modulation amplitude and d_0 the average tip height), by interpolating the scattered signal, $E^{\mathrm{sca}}(d)$, at a total of 100 positions in d. A tapping amplitude of 80 nm was assumed. The so-obtained complex-valued scattered signal, $E^{\mathrm{sca}}(d)$, was demodulated at the 3rd harmonic, 3Ω, to yield the calculated near-field signal, E_3^{raw}. Signal normalization was performed as it was done in the experiment by normalizing the demodulated spectra, E_3^{raw}, to the

reference signal, E^{ref}, yielding the normalized demodulated scattering signal $\tilde{s}_3 = s_3 e^{i\varphi_3} = E_3^{\mathrm{raw}}/E^{\mathrm{ref}}$. The corresponding normalized amplitude and phase spectra, $s_3(\omega)$ and $\varphi_3(\omega)$, are shown as solid curves in Fig. 3(b) in the main text.

For comparison with the calculations assuming a core-shell nanoparticle with an absorbing shell (PDMS), we repeated above calculation with a non-absorbing shell, where we assumed a background permittivity of $\varepsilon_{\mathrm{bkg}} = 1.8$ to match the non-resonant permittivity component of the Si-CH_3 vibrational resonance of PDMS, yielding the demodulated scattered field, $\tilde{s}_3^{\mathrm{bkg}}(\omega) = s_3^{\mathrm{bkg}}(\omega) e^{i\varphi_3^{\mathrm{bkg}}(\omega)}$ (shaded curves in Fig. 3(b) in the main text). With the help of these spectra, isolation of the spectral signature of the molecular vibration of the numerically calculated data could be done straightforwardly by taking the following difference: $\Delta s_3(\omega) = s_3(\omega) - s_3^{\mathrm{bkg}}(\omega)$ (isolating the spectral signature in amplitude) and $\Delta\varphi_3(\omega) = \varphi_3(\omega) - \varphi_3^{\mathrm{bkg}}(\omega)$ (isolating the spectral signature in phase). To perform this isolation with the experimental data, we used the calculated spectra, $s_3^{\mathrm{bkg}}(\omega) e^{i\varphi_3^{\mathrm{bkg}}(\omega)}$, as a substitute for experimental spectra of an AFM tip covered by a non-absorbing dielectric film (which would be difficult to prepare experimentally because of the difficulty to match precisely the thickness and non-resonant permittivity component of PDMS). To this end, we scaled the calculated spectra as follows. The calculated spectra assuming an absorbing PDMS shell, $s_3(\omega) e^{i\varphi_3(\omega)}$ (red line in Supplementary Fig. 5), were scaled to the maximum of the experimental spectra (black line). Using the same scaling factor, we then plotted the calculated spectra assuming a non-absorbing dielectric shell, $s_3^{\mathrm{bkg}}(\omega) e^{i\varphi_3^{\mathrm{bkg}}(\omega)}$ (dotted grey line in Supplementary Fig. 5 and shaded area in Fig. 3(a) in the main text). After this scaling, we could isolate the spectral signature of the molecular vibrations in the experimental data by straightforwardly calculate the differences, $\Delta s_3(\omega) = s_3(\omega) - s_3^{\mathrm{bkg}}(\omega)$ and $\Delta\varphi_3(\omega) = \varphi_3(\omega) - \varphi_3^{\mathrm{bkg}}(\omega)$.

To evaluate the field-enhanced molecular scattering, $\mathbf{E}_{\mathrm{AOA}}$, from the scattering model (Eqs. 18, 19 in the main text) (plotted as dashed lines in Fig. 3(b,g) in the main text), we calculated the field enhancement, $f \approx f_z$, for the unloaded nanorod antenna at the lowest position of the particle, *d*, where we only consider the *z*-component as it is the dominant component over the x- and y-components above the metal nanorod. Further, we evaluated the polarizability of the nanoparticle, α_{O}, by using the analytical model for a core-shell nanoparticle in the quasistatic limit[11]:

$$\alpha_{\mathrm{O}} = 4\pi R_2^3 \frac{R_2^3(\varepsilon_2 - 1)(\varepsilon_1 + 2\varepsilon_2) + R_1^3(\varepsilon_1 - \varepsilon_2)(1 + 2\varepsilon_2)}{R_2^3(\varepsilon_2 + 2)(\varepsilon_1 + 2\varepsilon_2) + 2R_1^3(\varepsilon_2 - 1)(\varepsilon_1 - \varepsilon_2)} , \qquad (1)$$

where we assumed a radius for the metallic core of $R_1 = 50$ nm and a 10 nm thick PDMS coating, i.e. $R_2 = 60$ nm, and permittivity for Au (ε_1) and PDMS (ε_2) as in the numerical calculation above.

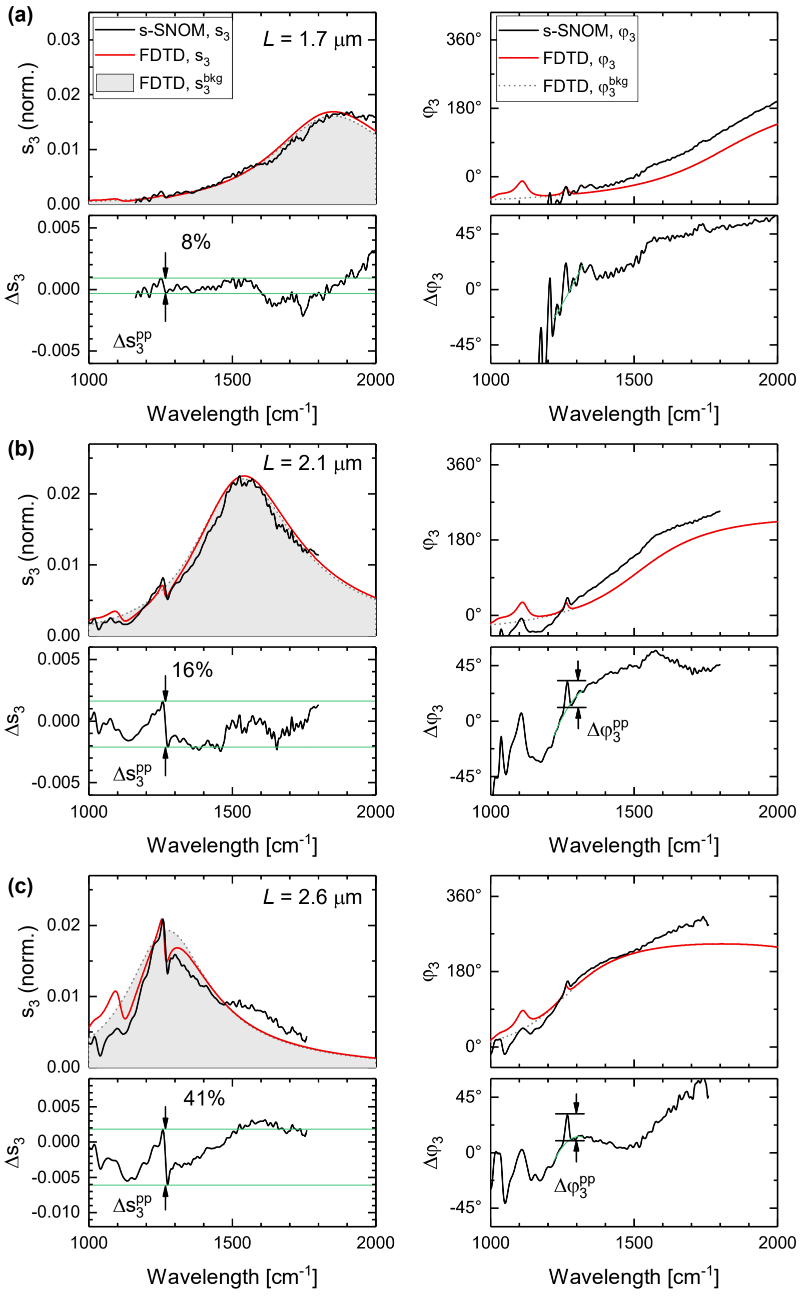

Supplementary Figure 5: Extraction of the spectral signature of the molecular vibration for Fig. 3 in the main text. (legend next page)

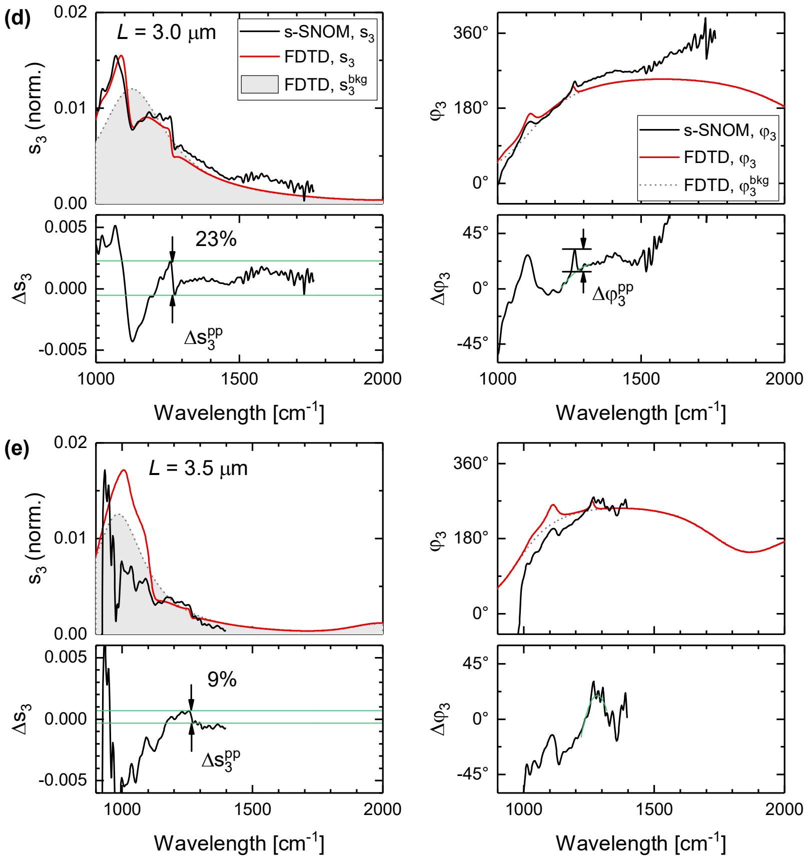

Supplementary Figure 5 (cnt'd): Extraction of the spectral signature of the molecular vibration for Fig. 3 in the main text. Spectra as obtained with the tip position at the left end of the nanorod. (a) Nanorod length, $L = 1.7$ μm, (b) $L = 2.1$ μm, (c) $L = 2.6$ μm, (d) $L = 3.0$ μm and (e) $L = 3.5$ μm. s_3 and φ_3: experimental amplitude and phase spectra obtained with a PDMS-contaminated AFM tip (s-SNOM, black line) and calculated spectra (FDTD, red line) obtained by assuming a Au core-PDMS shell nanoparticle. s_3^{bkg} and φ_3^{bkg}: numerically calculated amplitude and phase spectra assuming a non-absorbing dielectric shell (grey shaded area and gray dotted line). Bottom left panels: values indicate the contrast of the spectral signature of the 1,258 cm^{-1} (Si-CH_3) molecular vibration of PDMS relative to the maximum of the antenna scattering. Horizontal green lines denote the maximum and minimum of the spectral signature in s_3 (bottom left panel). The green curve in the bottom right panel denotes an estimation for the baseline in φ_3 (polynomial fit), which is taken as reference to determine the peak height.

Supplementary Note 2 – Near-Field images of the rod antennas

In Supplementary Fig. 6(a) we show spectrally integrated near-field maps of the rod antennas in Fig. 3 of the main text, as obtained by blocking the reference arm of the nano-FTIR interferometer and subsequently recording a near-field image. While the fundamental dipolar mode is observed on the rod antennas, as expected, importantly, the signal on the substrate is very small and below the noise floor. This was already observed for this setup in ref. [12]. Line profiles taken across the long axis of the rod quantity this observation (Supplementary Fig. 6(b)). Performing near-field spectroscopy on the antenna and on the substrate further reveals that the near-field signal is near zero on the substrate (Supplementary Fig. 6(c)). Therefore, we can conclude that (i) demodulation suppresses any direct contribution of the PDMS-layer and (ii) the vibrational features observed in Fig. 3 in the main text solely stem from the molecules (on the tip) scattering via the antenna after being illuminated by the antenna, i.e. the field-enhanced molecular scattering $\mathbf{E}_{\mathrm{AOA}}$.

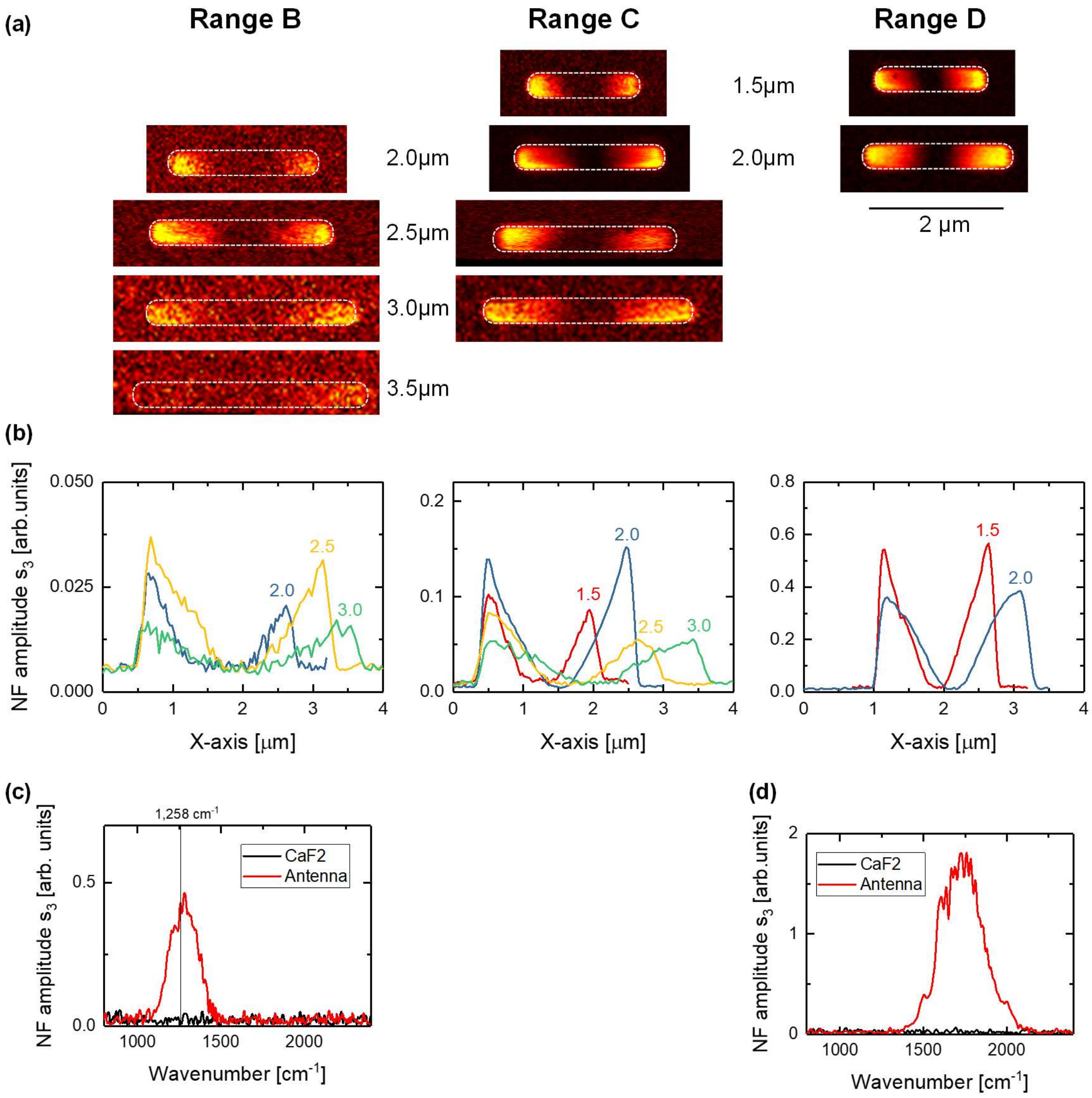

Supplementary Figure 6: White-light images of the rod antennas and other test measurements. (a) White-light images of the rod antennas taken at different settings of the nano-FTIR laser (B: 850 to 1450 cm^{-1}, C: 1200 to 1900 cm^{-1} and D: 1500 to 2200 cm^{-1}, rod antenna length is stated next to the images). The fundamental dipolar mode is clearly visible. Note that signal-to-noise is limited compared to the near-field spectra shown in Supplementary Fig. 5 owing to the short integration time. (b) Line profiles taken along the long rod axis in (a), averaged over the width of the rods. (c,d) Raw near-field amplitude spectra obtained on the antenna and on the adjacent CaF_2 substrate. A 2.5 µm long rod antenna was measured in (c) and a 1.8 µm rod antenna in (d).

References

1. Palik, E. D. *Handbook of Optical Constants of Solids*. (Academic Press, 1985).
2. Rezus, Y. L. A. & Selig, O. Impact of local-field effects on the plasmonic enhancement of vibrational signals by infrared nanoantennas. *Opt. Express* **24**, 12202 (2016).
3. Sun, J., Carney, P. S. & Schotland, J. C. Strong tip effects in near-field scanning optical tomography. *J. Appl. Phys.* **102**, 103103 (2007).
4. Chen, X. *et al.* Modern Scattering-Type Scanning Near-Field Optical Microscopy for Advanced Material Research. *Adv. Mater.* **31**, 1804774 (2019).
5. Lo, Y.-S. *et al.* Organic and Inorganic Contamination on Commercial AFM Cantilevers. *Langmuir* **15**, 6522–6526 (1999).
6. Jahng, J., Yang, H. & Lee, E. S. Substructure imaging of heterogeneous nanomaterials with enhanced refractive index contrast by using a functionalized tip in photoinduced force microscopy. *Light Sci. Appl.* **7**, 73 (2018).
7. Zhang, X., Qiu, J., Zhao, J., Li, X. & Liu, L. Complex refractive indices measurements of polymers in infrared bands. *J. Quant. Spectrosc. Radiat. Transf.* **252**, 107063 (2020).
8. Çapoğlu, I. R., Rogers, J. D., Taflove, A. & Backman, V. The Microscope in a Computer: Image Synthesis from Three-Dimensional Full-Vector Solutions of Maxwell's Equations at the Nanometer Scale. in *Progress in optics. Volume fifty seven Volume fifty seven* (ed. Wolf, E.) 1–91 (Elsevier, Oxford; New York, 2012).
9. Schnell, M. *et al.* High-resolution label-free imaging of tissue morphology with confocal phase microscopy. *Optica* **7**, 1173–1180 (2020).
10. Alonso-González, P. *et al.* Resolving the electromagnetic mechanism of surface-enhanced light scattering at single hot spots. *Nat. Commun.* **3**, 684 (2012).
11. Bohren, C. F. & Huffman, D. R. *Absorption and Scattering of Light by Small Particles*. (WILEY-VCH, 1998).
12. Virmani, D. *et al.* Amplitude- and Phase-Resolved Infrared Nanoimaging and Nanospectroscopy of Polaritons in a Liquid Environment. *Nano Lett.* **21**, 1360–1367 (2021).
